# Indents

**Published:** 1910-09-15

**Authors:** 


### INDENTS.

☞Brief Articles of Technical or Scientific Value will receive notice and comment. Contributions invited.

*The Chip Blower.* That first impressions are lasting is perhaps more of a truism in the dental office than anywhere else.

Imagine the impression a dentist makes upon the new or prospective patient, if about the first thing that is done is to take up a soiled looking old rubber bulb and blow a blast of sewer gas into the mouth, in making an examination of the occlusal surfaces of the buccal teeth.

Do not draw perfume into the instrument as one has suggested. This only adds insult to injury. Do not depend upon heating them over a bunsen.

Do not depend on having several different ones, giving each a time to dry and purify, as some one else has suggested. You will be sure to use the wrong one when that desirable party comes to your office, and you will be left to wonder why she never returned to have the work done.

Purchase one of the regulation chip blowers, remove the bulb, go down to the drug store and get a bulb with a metal intake valve in the back, put your new air nozzle into it and when once you become accustomed to its use, you will forget that you were ever annoyed with the old fetid unsanitary variety of instrument. Keep the bulb clean and white by occasionally washing it in ammonia water.—C. E. Allen, *Review.*

*New Method of Ether Anesthesia.* The method used by Hornabrook is one of open ethyl chlorid followed by open ether. The mask used is an ordinary wire frame, covered with one layer only of very thin flannel. The mask is placed on the patient's face, and the patient told to breathe quietly and naturally; that there will be no unpleasant sensation of suffocation, and that after a few drops of the anesthetic have been placed on the mask a pleasant floating sensation will come over him. The ethyl chlorid is dropped, not sprayed on to the mask. If ethyl chlorid is sprayed on, it freezes on the mask, does not act so well on the patient, and more is used than necessary; the drops should be rapid and spread over a wide area; from 3 to 8 c.c. are generally required. Hornabrook has never seen a sign of collapse under this open ethyl method, and in one case 25 c.c. were dropped on the mask to try its effect on the patient. The patient passes under the anesthetic rapidly and pleasantly, without any feeling of suffocation or discomfort. After 5 c.c. of ethyl chlorid have been dropped on the mask, a few minims of ether are dropped—that is in ordinary women or anemic men; if the patient is full-blooded or alcoholic, it is better to pass from ethyl chlorid to chloroform before going on with the ether. As a rule not more than 30 minims at the most of chloroform are necessary before muscular relaxation is obtained, and ether can then be substituted. Muscular relaxation when once obtained does not return to rigidity except in very rare instances, and even if it does, a few drops of chloroform will reduce it. The time taken to place the patient under anesthesia ready for the knife is from three to five minutes from the first drops of ethyl chlorid. A patient can be maintained under this open ether for any length of time. There is practically no secretion of mucus, so that the air passages remain clear; the breathing is quiet and easy; the color of the patient remains excellent, it being very rare to see blueness, even in prolonged cases; the patient recovers from the anesthetic very quickly; the pupils should be maintained very active and of medium size throughout; and the upper lid should remain elastic to the touch. Care must be taken that the anesthetist has two masks, for after a time a coating of moisture forms on the under surface of the flannel and this acts as a barrier between the patient and the ether, so that the patient gets none or very little ether in spite of the quantity poured on the mask, and the patient then commences to come out. The mask should be changed for a dry one before this happens, and the old one wiped free from moisture to be used again later; it may be necessary in a prolonged operation to change the mask more than once. The amount of ether poured on the mask varies with the length of the operation, but, speaking generally, about 20 minims every half minute, the quantity used is from 4 to 5 ounces in the first hour and less in the second. The state of anesthesia induced is light, the muscular relaxation good, and even should there be rigidity, as may be the case, it is easily overcome with a few drops of chloroform. The patient can be brought out of the anesthesia very easily and rapidly, in fact, in many respects it resembles sleep rather than deep narcosis of an anesthetic, the shock of even a very prolonged and severe operation is well-borne, the patient being in no way depressed as he would have been under chloroform, or irritated by concentrated ether vapor and excessive secretions of mucus as under a closed method.—*A. M. A. Journal.*

*Surgical Treatment of Chronic Abscesses.* When a chronic abscess arises showing a sinus, investigation is made, first, with a soft abscess probe. After the apex of the root has been sighted, a drop or two of a 1 per cent solution of cocain is injected into the pocket, after which a small spoon excavator is used to scrape the infected root, and all the necrosed territory embraced by the pocket. After this has been thoroughly done and all the serumal deposits removed, a few drops of an aromatic sulphuric acid compound is introduced with a Dunn syringe and the case dismissed without further treatment for a week. If the sinus shows nothing further and if the root canal has been thoroughly treated and filled there will probably be no recurrence of the abscess.

The aromatic sulphuric acid compound mentioned above is as follows:

Aromatic Sulphuric Acid, 3iii, and Tincture of Capsicum drops x, parts 70; Oil of Cinnamon, 10 parts; Formaline, 5 parts; Alcohol, 15 parts. Sigh–Use full strength.

—G. A. Billow, in *Summary.*

*Antrum Treatment.* Nasal surgeons do not magnify the difficulty of getting into the antrum. We can get into the antrum as readily as you can. The point is not the ease with which you can get into the antrum, but to find the cause of the antrum trouble and determine the proper operation. If you find it in the nose, it is folly to attack the mouth; if you find it in the mouth, it is folly to attack the nose; but the investigations of men like Cryer of Philadelphia, Fletcher of Cincinnati, Talbot of Chicago, men who are orthodontists certainly are not biased. If they were biased, they would be biased in favor of the mouth being the seat of the disorders, but the testimony of these men, as I read it, is in line distinctly with that of the most advanced rhinologists—that is, in a large majority of cases the maxillary antrum trouble originates in the nose. A certain small percentage, about 11 to 14 per cent do originate from caries of the teeth. Now it is not, that you see cases more often from one cause, and I more often from another, but the cases have been analyzed by men of a very judicial, unbiased mind, and their decision is in line with one another. I do not think we differ so much from one another. No one denies, for instance, that caries may cause sinus trouble. I want to say further that diseases of the maxillary antrum have been shown, by a great number of men, notably Cryer, to be due to caries of teeth, in a *very small* percentage of cases. Formerly the assumption was that maxillary antrum suppuration was almost invariably due to caries of the teeth. We now know that various exanthemata—grippe, measles, typhoid fever, scarlet fever—largely affect the maxillary antrum. If you find a maxillary sinus with a great alveolar process and the trouble originating in the frontal sinus, what in the name of common sense is the use of opening anywhere but the most important point, and attacking it from the frontal sinus? The methods of opening the antrum, there are many ways. The question is to locate the proper method for the proper case. I do not treat two cases of maxillary antrum, possibly, alike. If I find the maxillary antrum by means of my X-Ray to be divided by a septum and the frontal sinus and ethmoidal sinus perfectly normal, what in the name of common sense is the use of attacking the ethmoidal sinus. We know that we take backward children, and I do not mean to say that every backward child has adenoids, or that every backward child has mal-occlusion or eye-strain, but we are physicians and we should seek every possible cause which might influence this. Dullness, listlessness is due to interference with the proper lymphatic circulation in the lymphatics of the meninges by reason of hypertrophic growth interfering with cerebral circulation; if it is eye-strain in one case, correct that; if mal-occlusion, correct that, because we know that if the child does not masticate properly he cannot receive proper nourishment and cannot get on; if it has adenoids, remove them. Where you find adenoids which are the fault of backward children, removing the adenoids cures them, there is no doubt about that, and we know also that there have been adenoids in children long before we have done adenoid operations, but we recognize that great harm was done to their hearing, their breathing, their chewing, etc., and we hope to end these difficulties in the rising generation. I have operated on children in the families of some of the men who are criticising this operation and they know what great benefits come. It is well to know that we operate for adenoids even in infants, but you should know that we do not attempt thorough removal before the third year, as the adenoids are necessary then for the proper functioning of the eustachian tube in the very young: where anatomical reasons influence this course.—Dr. E. J. Bernstein in *Summary*.

*The Management of Patients in General.* As has already been intimated, no dentist can expect to be successful unless he first learns how to manage the different types of people who come to him. A dentist should be able to read character in the face of every individual and to know how to approach each different class the moment they appear. Not that he should constantly sink his own individuality into that of others, but that he may so control the individualities of those who come under his ministrations that he can make his practice run along smoothly and without the friction which often mars the career of the most promising practitioners. Enough has already been said along this line, but its importance cannot be too strongly urged nor too often repeated.—Dr. C. N. Johnson in *Summary*.

*Extraction of Cuspids.* My method of extracting upper cuspids, these being usually the last teeth to come out, and if lateral and bicuspids are present, I remove them, thus leaving the cuspid standing alone, and instead of grasping the root on its lingual and labial sides, I use a S. S. W. No. 10 bayonet forcep and grasp the root on the mesial and distal sides, they being slightly flat, and the process dips back, allows the beaks to slip high on the root, then grasp handles with both hands and rotate either way just enough to laxate the tooth, pressing upward while doing this and the root will come away clean, even if there is a decided curve at the apex, and requires simply a good grip in the hand doesn't break the process any, there is no shock.

For broken down roots that can't be grasped with forceps I use a No. 11 S. S. W. gouge or spade which I have hollowed out on the flat side, making it concave,this being small and then I am able to insert it between the root and process high up, then with a little twist or rotating, the root loosens, when you can pick it out; have taken out bad ones either upper or lower third molars, even partial impact also.—J. A. Coates, in *Summary*.

*Wax Dummies.* To make your wax dummies for cast bridge work, take any of the dieplates where you have the bicuspids and molars depressed, select the one or ones to suit your case. Then having the mould wet you can press the warm inlay wax to place, getting cusp and body; which can be trimmed to suit both the model and articulation, with but little trouble. Making each dummy separate you can articulate, and join together with a warm spatula to cast, or cast separate.—E. B. Hill in *Summary*.

*Tooth Powder*

℞

| | |
|---|---|
| Pulv Castile Soap | gr. 90 |
| Pulv Sacch Alba | ʒ ij |
| Tin Oxide (Merks White) | ℥ j |
| Chinosal | gr. ij |
| Creta Precip | ℥ iij |
| Flavor, q. s. | |

Sig.—Once a day.

Jungman.

*For Relief of Toothache.*

℞

| | |
|---|---|
| Creosote | |
| Chloroform | |
| Oil of Cloves, āā | pts. 10 |
| Camphor | " 7 |
| Phenol | " 2½ |

Sig.—Apply to pulp of tooth, on lint. Sure relief.

*Law.* Dr. Robinson has made a statement in his report which should not pass unanswered. I refer to his statement "That the Dental Surgeon in the State of Pennsylvania does not possess the right to give a prescription to a patient which the druggist is required by law to fill" and that he "denies the right of a dentist to medically treat a tooth."

In answer to these statements I would refer you to Williams "On Laws Relating to Physicians, Druggists and

Dentists" and to an article by Frederick Worthen Frost, A. B., L. L. B., an eminent member of the New York Bar, in the March number of the *Dental Brief*, volume XIV, entitled "Some Legal Questions Involved in the Practice of Dentistry."

Each of these authorities states "that the law relating to dentistry is, for many purposes, simply one branch of the general law relating to physicians and surgeons. That the word physician includes in law all who practice physic or surgery and is not limited to any one school of practitioners. That, in the broad sense, the term physician includes a dentist. That along broad general lines, for all practical purposes, the law applies alike to physicians, surgeons and dentists. That by merely undertaking the treatment of a patient, the physician (and this includes dentists), implies by contract with the patient that he has such skill, science and information as will enable him properly and judiciously to perform the duties of his profession. A dentist undertaking the treatment of a patient is only required to possess and exercise that degree of skill and learning ordinarily possessed and exercised by the members of his profession in good standing, practicing in similar localities, and it is his duty to use reasonable care and diligence in the exercise of his skill and the application of his learning, and to act according to his best judgment. One who holds himself as a specialist in the treatment of particular diseases or conditions is bound to exercise such skill and to possess such learning as is exercised and possessed by practitioners who are specialists in the treatment of such diseases or conditions."

The druggist does not necessarily have to fill a prescription from any pratitioner, whether he be a physician, surgeon or dentist. Neither does a dentist have to treat a patient unless he has already undertaken the case.

In certain states there are special statutes governing the dispensing of medicines or drugs. In some instances these limit the rights of the dentist, but broadly speaking, and especially in Pennsylvania, the legal rights of the dentist to prescribe are the same as those of the general physician, except as limited by the diseases which he has a right to treat. There is no question as to the right of the dentist to treat diseases of the oral cavity and to prescribe for the same.—W. L. Fickes in *Summary*.

*Porcelain Facings.* Having properly prepared your root fit Dowel pin or post as near the shape of the canal as possible, allowing it to extend a little beyond the root.

Now select a White or Davis detached post crown as near as possible the length desired, grind flat on the posterior surface until a little more than one-half of the post hole remains, holding it at such an angle that the incisal edge will be ground down to a point. The grinding should be done by holding the facing on the flat side of a perfect stone. Place pin in position in root. With warm inlay wax press facing, which has previously been coated with vaseline, to place; chill and remove. Post should pull out in place; carve and polish. Remove facing, insert sprue and cast.

This method may be used for dummies and can if desired be cast in one piece, although I prefer soldering. This will not only give you a stronger facing, but more bulk of porcelain, consequently the color will not be impaired.—D. H. Carpenter in *Summary*.

### ANNOUNCEMENTS.

Oral Surgery. Dr. Truman W. Brophy is writing a book on Oral Surgery which will be published this fall by P. Blakeston's Son & Co. Dr. Brophy has the technical skill and experience of many years of teaching this subject, and

